# Periostin involved in the activated hepatic stellate cells-induced progression of residual hepatocellular carcinoma after sublethal heat treatment: its role and potential for therapeutic inhibition

**DOI:** 10.1186/s12967-018-1676-3

**Published:** 2018-11-06

**Authors:** Rui Zhang, Xia-Hui Lin, Min Ma, Jie Chen, Jun Chen, Dong-Mei Gao, Jie-Feng Cui, Rong-Xin Chen

**Affiliations:** Liver Cancer Institute, Zhongshan Hospital, Fudan University and Key Laboratory of Carcinogenesis and Cancer Invasion, Ministry of Education, Shanghai, China

**Keywords:** Hepatocellular carcinoma, Hepatic stellate cells, Periostin, Calcipotriol

## Abstract

**Background:**

Incomplete thermal ablation may induce invasiveness of hepatocellular carcinoma (HCC). Here, we investigated whether activated hepatic stellate cells (HSCs) would accelerate the progression of residual HCC after sublethal heat treatment, and thus sought to identify the potential targets.

**Methods:**

Hepatocellular carcinoma cells were exposed to sublethal heat treatment and then cultured with the conditioned medium from activated HSCs (HSC-CM). The cell proliferation, migration, invasion and parameters of epithelial–mesenchymal transition (EMT) were analyzed. In vivo tumor progression of heat-treated residual HCC cells inoculated with activated HSCs was studied in nude mice.

**Results:**

HSC-CM significantly enhanced the proliferation, motility, invasion, prominent EMT activation and decreased apoptosis of heat-exposed residual HCC cells. These increased malignant phenotypes were markedly attenuated by neutralizing periostin (POSTN) in HSC-CM. Furthermore, exogenous POSTN administration exerted the similar effects of HSC-CM on heat-treated residual HCC cells. POSTN induced the prominent activation of p52Shc and ERK1/2 via integrin β1 in heat-exposed residual HCC cells. Vitamin D analog calcipotriol blocked POSTN secretion from activated HSCs. Calcipotriol plus cisplatin significantly suppressed the activated HSCs-enhanced tumor progression of heat-treated residual HCC cells via the inhibited POSTN expression and the increased apoptosis.

**Conclusions:**

Activated HSCs promote the tumor progression of heat-treated residual HCC through the release of POSTN, which could be inhibited by calcipotriol. Calcipotriol plus cisplatin could be used to thwart the accelerated progression of residual HCC after suboptimal heat treatment.

**Electronic supplementary material:**

The online version of this article (10.1186/s12967-018-1676-3) contains supplementary material, which is available to authorized users.

## Background

Radiofrequency ablation (RFA) has been established as the standard care for unresectable early hepatocellular carcinoma (HCC) with complete response rates exceeding 90% [1, 2]. Due to its superiorities such as effectiveness, minimal invasiveness, safety and repeatability, RFA has been advocated to treat the patients with medium-large HCC [3, 4]. However, RFA efficacy diminishes and local tumor recurrence increases (10–52.4%, depending on tumor size) [4–6], which greatly impairs survival of patients. When RFA is used to treat HCC beyond 3 cm, local tumor recurrence is a common phenomenon because it is difficult for RFA to secure a sufficient safety margin in three dimensions to ablate microsatellite or microvascular invasion around the tumor [7]. So, local tumor recurrence arises from those minimal or occult residual tumors. Even more, rapid and aggressive tumor progression after incomplete RFA has been increasingly reported [8–13], albeit its mechanisms are not fully understood.

Previous studies have shown that the change of HCC cells in response to heat stress, such as sarcomatous appearance, heat-resistant subline, enrichment of cancer stem cells or occurrence of epithelial–mesenchymal transition (EMT) [14–19], is associated with accelerated tumor progression after suboptimal heat treatment. However, these studies neglect the potent signals from tumor microenvironment that may favor tumor progression.

Hepatocellular carcinoma occurs within a fibrotic tumor microenvironment rich in activated hepatic stellate cells (HSCs). Evidences have shown that mutual interactions between HCC and activated HSCs promote the tumorigenicity, growth, migration, invasion, angiogenesis and metastasis of HCC [20–24]. More importantly, Rozenblum et al. observed the mass accumulation of the activated HSCs at the perimeter of the ablation zone [25]. This prompted us to hypothesize that the cross-talks between activated HSCs and residual HCC cells would enhance the tumor progression after incomplete thermal ablation and they could be potential therapeutic targets.

Here, we provided the evidence how activated HSCs enhanced tumor progression of heat-treated residual HCC as the following: (i) activated HSCs-derived condition medium (HSC-CM) aggravated malignant phenotypes of heat-exposed residual HCC cells *(*ii) periostin (POSTN) mediated the tumor-promoting effects of HSC-CM (iii) vitamin D analog calcipotriol blocked POSTN secretion from activated HSCs (iv) calcipotriol plus cisplatin suppressed the in vivo activated HSCs-promoted tumor progression of the residual HCC cells via inhibition of POSTN expression and an increase of apoptosis.

## Methods

### Cell culture and conditioned medium (CM) collection

HCC cell lines MHCC97H and MHCC97H with integrin-β1 knockdown (Liver Cancer Institute of Fudan University, Shanghai, China), Hep3B and HepG2 (ATCC, USA), Huh7 (Japanese Cancer Research Bank) were grown in DMEM with 10% FBS (Gibco) and 1% penicillin/streptomycin. Cell lines were authenticated by short tandem repeat validation analysis during the study period. Primary human hepatic stellate cells (pHSCs) (Sciencell, USA) were maintained in the provided medium and LX2 cells (a gift from S. Friedman) were cultured in DMEM with 2% FBS. All cell cultures were carried out in a 37 °C incubator with a humidified atmosphere in presence of 5% CO_2_.

As in our previous description [26], conditioned medium was collected from activated HSCs (HSC-CM) and anti-human POSTN antibody (2.5 μg/mL) (Abcam, Cambridge, UK) was added into HSC-CM to neutralize the activity of POSTN.

To obtain conditioned medium from calcipotriol-treated HSCs, pHSCs or LX2 cells were pre-stimulated using 10 ng/mL TGF-β1 and then incubated with 100 nM calcipotriol (Sigma-Aldrich) for 12 h, replenished with fresh medium for another 24 h and the medium was collected for the subsequent experiments.

### In vitro heat treatment

As previously described [26], HCC cells were heated at the pre-set temperatures, seeded into 96-well plates and cell viability was measured at 48 h after heat treatment. IT50 was calculated to indicate the temperature of inducing a 50% reduction in cell viability compared with the 37 °C control. The IT50 data was used to simulate in vivo sublethal heat condition.

### Cell proliferation

Cell proliferation was measured using the cell viability assay WST-1 or BrdU ELISA kit (Roche, Manheim, Germany) according to the manufacturer’s instructions. Briefly, HCC cells were subjected to sublethal heat treatment and plated in 96-well plates (8 × 10^3^ cells/well). Equivalent amount of HSC-CM or control medium was added into each well and replaced daily to remove cell debris. The absorbance was measured at 450 nm on a multiskan spectrum reader (Thermo Scientific).

Cell co-culture were performed using a transwell system containing the 0.4 μm pore filter insert (Millipore, Switzerland) allowing medium components freely diffusion but restricting cells migration. Briefly, heat-treated HCC cells were plated in the bottom alone or together with HSCs cells in the transwell insert (4:1 ratio). After 4 days, the proliferation of HCC cells in the bottom wells were measured using WST-1 reagent.

### Cell migration and invasion assay

As previously described [27], migration and invasion assays were carried out. Briefly, in the migration assay, heat-treated residual HCC cells (1 × 10^5^) were seeded into the upper chamber while 600 μL HSC-CM or control medium was added into the lower chamber, and then incubated for 36 h. The invasion assay was done in a similar manner except transwell insert pre-coated with Matrigel (BD Biosciences) and culture for 72 h. At the end of experiments, the cells in the upper chamber were removed with cotton swabs and the cells on the lower surface of the inserts were fixed, stained and photographed from five random fields (100× magnification) under phase contrast microscope (Leica, Germany).

### Colony formation assay

After sublethal heat treatment, HCC cells (2 × 10^3^ cells) were seeded into 6-well plates and equal volume of HSC-CM or control medium was then added into each well for 8 days. Cell colony with a diameter larger than 50 μm was counted using ImageJ software (National Institutes of Health, Bethesda, MD, USA) after fixed with methanol, stained with crystal violet and photographed.

### Flow cytometry analysis

Cell apoptosis was detected using annexin V staining kit (Invitrogen, Carlsbad, California, USA) according to the manufacturer’s instruction. In brief, heat-treated HCC cells were incubated with HSC-CM or control medium for 3 days. Then, cells were harvested and resuspended in annexin-binding buffer (1 × 10^6^ cells/mL). Subsequently, appropriate amount of Alexa Fluor 488 annexin V and PI working solution were added. Early and late apoptosis rates were analyzed by FACS Calibur flow cytometer (BD Biosciences, USA) and FlowJo software (Tree Star Inc, Ashland, Ore).

### Quantitative reverse transcription-PCR (qRT-PCR)

Total RNA was extracted using TRIzol reagent, transcribed and amplified with the use of RevertAid First Strand cDNA Synthesis kit and the Maxima SYBR Green qPCR Master Mix kit (Thermo Fisher Scientific, Waltham, MA, USA). The relative gene expression was calculated using the equation 2^−ΔΔCt^ where the Ct value of GAPDH or β-actin was used as the normalization. The PCR primers are shown in Additional file 1: Table S1.

### Western blot analysis

Western blot was performed as the previously described [26]. Briefly, total protein was extracted using RIPA buffer added with PMSF and phosphatase inhibitors. Protein concentration was determined using BCA protein assay (Millipore, Switzerland). Then, 20 μg of protein was separated by SDS-PAGE and transferred to polyvinylidene difluoride membranes (PVDF, Millipore, USA). After blocked with 5% non-fat milk, the membrane was incubated with primary antibody against PCNA (1:2000), Snail (1:1000), Vimentin (1:1000), E-cadherin (1:1000), N-cadherin (1:1000), integrin β1 (1:1000), Shc (1:1000), phosopho-Shc (1:2000), ERK1/2 (1:1000) or phosopho-ERK1/2 (Thr202/Tyr204) (1:2000) (Cell Signaling Technology, USA), periostin (1:1000), collagen I (1:5000), α-SMA (1:300) or anti-vitamin D receptor antibody (1:1000) (Abcam, USA), GAPDH (1:1000), tubulin (1:1000) or β-actin (1:1000) (Beyotime, China) at 4 °C overnight and the corresponding HRP-conjugated secondary antibody for 1 h at room temperature on the next day. The membrane was developed with enhanced chemiluminescence (ECL; New Cell & Molecular Biotech Co., China).

### Immunohistochemical analysis

As previously described [28], immunohistochemistry was conducted using the streptavidin–peroxidase two-step method. Briefly, tissue section was deparaffinised, rehydrated, heated with antigen retrieval, blocked with 3% hydrogen peroxide and then incubated with primary antibody against POSTN (1:200), α-SMA (1:100), PCNA (1:400), cleaved-Caspase-3 (1:100) or E-cadherin (1:100) (Cell Signaling Technology, USA) at 4 °C overnight. The immunoreactivity and nuclear counterstain was performed using EnVision two-step visualization system (GeneTech, Shanghai, China) and hematoxylin. Photographs of three representative fields (200× magnification) were captured by microscope (Leica, Germany).

### Dual luciferase assay

The 1284-base pair cDNA containing the coding region of vitamin D receptor (VDR), named GV141-VDR, was purchased from Genechem (Shanghai, China). A luciferase reporter construct plasmid GV238 that contained the 5ʹ-flanking region of the POSTN gene from − 2000 to − 1 relative to the transcription start site (corresponds to − 2000 relative to the first AGA of 5′ UTR, amplified with the primers 5′-GATAGGTACCGCAAAGAACGACTAGGTTAAAATTG-3′ and 5′-AGATCTCGAGGAACTCTTTCCAGGAAGCATCGG-3′), as well as a series of deletion constructs (deleted at intervals of 500 base pairs from the 5ʹ end), were generated by GeneChem (Shanghai, China). Based on the conserved motif of VDR, the JASPAR database (http://jaspar.genereg.net/) was used to predict the binding sites of VDR on POSTN promoter sequence. Two putative sites in the promoter sequence of POSTN were identified (− 1829 to − 1815 and − 1415 to − 1401). To determine functionally relevant VDR binding sites, the putative binding sites for the seed region of VDR were mutated using site-specific sequence mutagenesis (CCCCACGGTTTCCAT). All promoter constructs were sequenced. HEK 293T cells were seeded into 24-well plates at 50–60% confluence per well. Cells were transfected with 1 μg POSTN promoter plasmids containing the firefly luciferase reporter, 1 μg VDR expression vector GV141-VDR and 20 ng Renilla luciferase expressing construct (as an internal control) into HEK 293T cells using Lipofectamine 2000 reagent (Invitrogen, CA, USA) and according to the manufacturer’s protocol, and cells were dealt with 100 nM calcipotriol for 12 h after 12 h of transfection. Twenty-four hours after transfection, the cells were detected for luciferase activity using the Dual-Luciferase Reporter Assay System (Promega, Madison, WI, USA) and multi-plate reader. Firefly luciferase activity is normalized to the Renilla luciferase activity. Each experiment was independently performed at least two times in triplicate.

### Gene expression profile

Total RNA was extracted and purified from heat-exposed residual MHCC97H cells treated with or without 100 ng/mL POSTN using miRNeasy Mini kit (QIAGEN, Germany). Genome-wide expression profiling was conducted using Affymetrix GeneChip Human Genome U133 Plus 2.0 Array according to the manufacturer’s instructions. Briefly, total RNA samples were amplified, labelled and purified to obtain biotin-labeled cRNA using GeneChip 3′IVT PLUS reagent Kit (Affymetrix, CA, US). The arrays were hybridized, washed and stained using the GeneChip Hybridization Wash and Stain Kit, and then scanned with a GeneChip Scanner 3000 (Affymetrix, CA, US) for subsequent generation of raw data. The gene expression data were processed and further analyzed using MAS 5.0 algorithm, Affy packages in R. Genes significantly differentially expressed were selected based on fold change > 2 or < 0.5, and T-test with P < 0.05. Gene annotation was based on Gene Ontology analysis. KEGG pathway enrichment analysis was conducted to examine the functional association between differentially expressed genes and to generate the significant gene networks. Differentially expressed genes were mapped to STRING database (http://string-db.org/) and then imported into Cytoscape software (http://www.cytoscape.org/) to acquire protein–protein interaction (PPI) network. The microarray data have been deposited into Gene Expression Omnibus (GEO) database (GSE108853).

Gene expression profiles of HCC cohorts (374 tumoral samples and 50 normal control samples) were downloaded from Genomic Data Commons Data Portal (GDC, https://gdc.cancer.gov/). Data processing and analysis were performed by using R Statistical Software. The expression enrichment of a gene signature was assessed significance for differences between the two groups using unpaired two-tailed Student’s t-test. The strength of associations between two parameters was assessed using Spearman’s correlation test. Survival plots were generated using Kaplan–Meier analyses and splitting the tumor samples into high- and low-expression groups. The median of tumor tissues was token as the threshold and significance for differences of survival between both groups was assessed by a log-rank test.

### Animal experiments

All animal experiments were conducted according to the guidelines of the Shanghai Medical Experimental Animal Care Commission and approved by the Ethical Committee on Animal Experiments of Fudan University, Shanghai, China.

Tumorigenicity assay was performed as described [29]. In brief, 2 × 10^4^ heat-treated residual MHCC 97H cells resuspended in 50% growth-factor-reduced Matrigel (BD Biosciences) were subcutaneously injected into the flanks of NOD/SCID mice alone or with 100 ng/mL POSTN. Mice were sacrificed 2 months post injection, at which time tumors were harvested.

As we previously described [26], 4-week old BALB/c nude mice were inoculated subcutaneously with heat-treated residual MHCC 97H cells (2 × 10^7^, n = 6) with pHSCs (ratio, 4:1) into the flanks of mice. Eighteen tumor-bearing mice were randomly assigned into three treatment groups: saline, calcipotriol (60 μg/kg, intraperitoneal, daily), or calcipotriol (60 μg/kg, intraperitoneal, daily) + cisplatin (4 mg/kg, twice a week). After 2 weeks, mice were sacrificed and tumor samples were harvested.

To examine the presence of activated HSCs in the tumors, CFSE labelled pHSCs (5 × 10^6^) with heat-treated residual MHCC 97H cells were inoculated subcutaneously into nude mice. At 2 weeks post injection, mice were euthanized and tumors were cryosectioned to detect the HSCs using fluorescence microscope (Leica, Germany).

### Statistical analysis

All statistical calculations were performed using Prism 6.0 (GraphPad Software, Inc., La Jolla, CA). Data were expressed as either mean ± standard deviation (SD) or percentage (%) when appropriate. Continuous data were analyzed by unpaired Student’s t-test or ANOVA between two groups or multiple groups whereas categorical data were compared using Chi-square test or Fisher’s exact test. Differences were considered statistically significant when *P* < 0.05 (two-side).

## Results

### HSC-CM promotes proliferation, EMT, colony formation and decreases apoptosis of heat-treated residual HCC cells

**Fig. 1:**
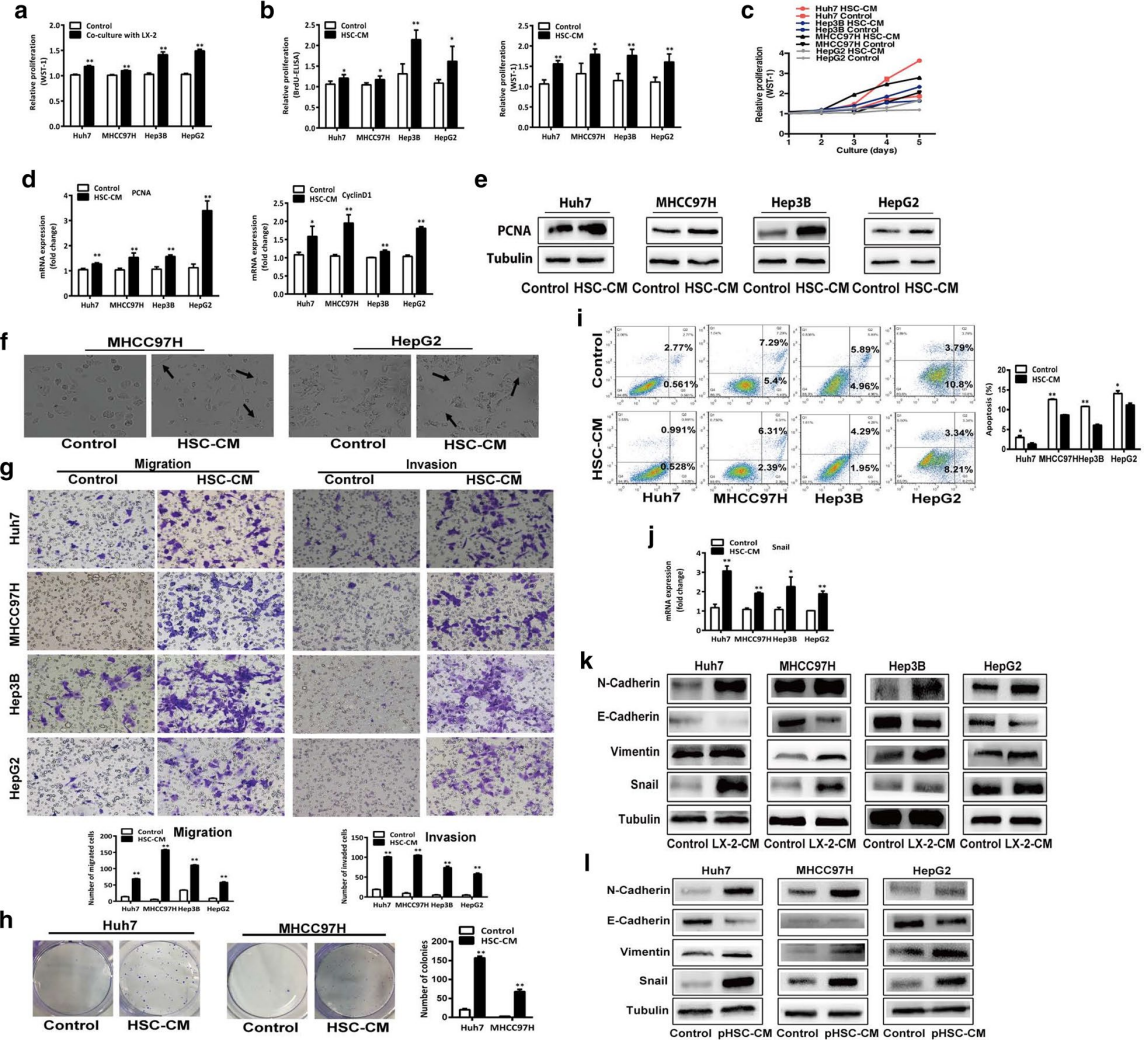
Activated HSCs or HSC-CM promoted the proliferation, migration, invasion, colony formation, and decreased the apoptosis of heat-exposed residual HCC cells. **a** When heat-treated residual HCC cells (Huh7, MHCC97H, Hep3B and HepG2) were co-cultured with activated HSCs LX2, proliferation was significantly higher than the cells cultured alone, as analyzed by WST-1 assay. **b**, **c** Proliferation of heat-treated residual HCC cells cultured in HSC-CM from LX2 cells was significantly higher than the cells cultured with control medium, as indicated by the WST-1 assay, BrdU-ELISA and growth curves. **d** PCNA and cyclinD1 mRNA expression of heat-treated residual HCC cells cultured in HSC-CM from LX2 cells were significantly higher than the cells cultured with control medium, as measured by qRT-PCR. **e** PCNA protein expression was analyzed by western blot. **f** Heat-treated residual HCC cells displayed distinct spindle-like appearance when cultured with HSC-CM from LX2 cells. **g** HSC-CM from LX2 cells promoted the in vitro migration and invasion of heat-treated residual HCC cells. The number of migrated and invaded cells was counted. **h** Colony formation of heat-treated residual HCC cells cultured in HSC-CM from LX2 cells was increased. **i** Apoptosis of heat-treated residual HCC cells in the presence of HSC-CM from LX2 cells was reduced than the cell cultured with control medium, as analyzed by Annexin V/PI staining. **j**–**l** The levels of Snail mRNA expression, and Snail, Vimentin, E-cadherin, and N-cadherin protein expression in heat-treated residual HCC cells cultured with HSC-CM from LX2 cells or pHSC cells compared with control medium were analyzed by qRT-PCR and western blot. *pHSC* primary hepatic stellate cells. **P < 0.01; *P < 0.05

As we previously described [26], 47.0 °C was chosen as IT50 to simulate the sublethal temperature at the tumor periphery during incomplete RFA. When HCC cells were subjected to sublethal heat treatment (47.0 °C for 10 min) and then co-cultured with activated HSCs or HSC-CM, cell proliferation was significantly higher than that of the cells cultured alone or with control medium (Fig. 1a–c). This was paralleled with the significant increase of proliferation-related transcripts (PCNA and cyclin D1) (Fig. 1d) and confirmed by Western blot for PCNA (Fig. 1e). After cultured in HSC-CM, heat-treated residual HCC cells showed a spindle-like appearance (Fig. 1f). As shown in Fig. 1g, HSC-CM significantly enhanced the in vitro migration and invasiveness of heat-exposed residual HCC cells. Compared with the cells cultured in control medium, colony formation efficiency of heat-exposed residual HCC cells in HSC-CM was significantly increased (Fig. 1h). In the presence of HSC-CM, heat-treated residual HCC cells survived more (Fig. 1i). Paralleled with these changes of cell phenotypes, the expression of EMT-related markers (N-cadherin, Vimentin and Snail) was significantly increased whereas E-cadherin was reduced (Fig. 1j–l). These data show that HSC-CM induces the more malignant and invasive phenotypes of heat-treated residual HCC cells by enhancing the proliferation, migration and invasion, colonic formation, EMT and reducing cell apoptosis.

### Periostin (POSTN) mediates the tumor-promoting effects of HSC-CM

Periostin secreted by fibroblast-like cells such as activated HSCs has been implicated in the progression of tumors [30, 31]. In our previous report [26], we found that POSTN expression in activated HSCs was significantly higher than that in HCC cells.

**Fig. 2:**
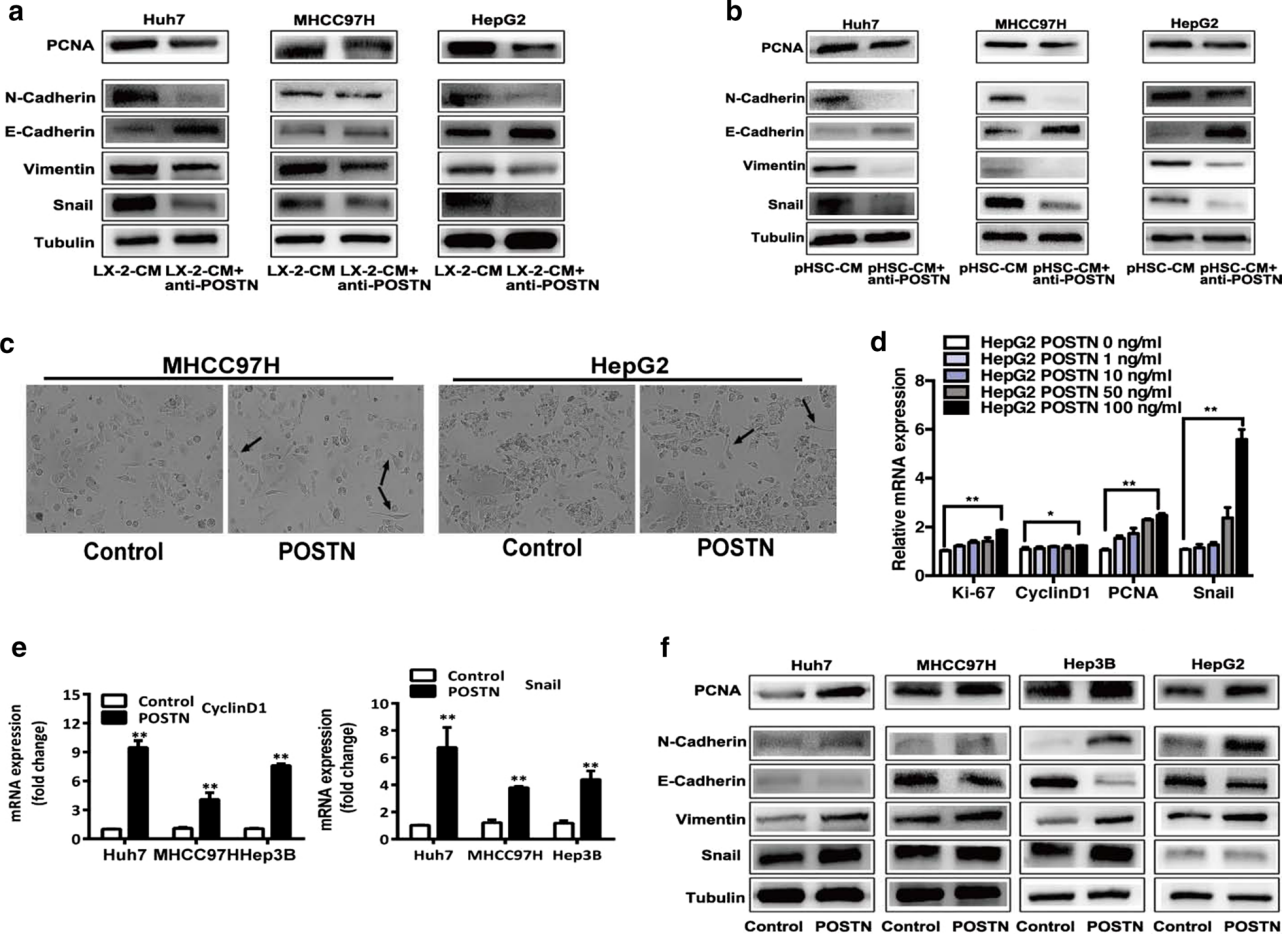
POSTN in HSC-CM mediated the increased proliferation, migration, and invasion of heat-exposed residual HCC cells. **a**, **b** The alterations of Snail, Vimentin, E-cadherin, N-cadherin, PCNA protein expression in heat-exposed residual HCC cells cultured with POSTN-depleting HSC-CM from LX2 cells or pHSC cells. **c** Distinct spindle-like appearance of heat-treated residual HCC cells (MHCC97H and HepG2) induced by exogenous POSTN (100 ng/mL). **d** After treated with exogenous POSTN, heat-exposed residual HepG2 cells showed significantly higher levels of Ki-67, cyclinD1, PCNA, Snail mRNA expression in a dose-dependent manner, as measured by qRT-PCR. **e**, **f** After treated with exogenous POSTN, heat-exposed residual HCC cells (Huh7, MHCC97H, HepG2 and Hep3B) showed significantly higher levels of cyclinD1, Snail, PCNA, N-cadherin, Vimentin and markedly lower level of E-cadherin compared to the control, as detected by qRT-PCR and western blot. *pHSC* primary hepatic stellate cells. **P < 0.01; *P < 0.05

To determine the crucial role of POSTN in HSC-CM, anti-POSTN antibody was added to neutralize the activity of POSTN. In the presence of anti-POSTN antibody, the effects of HSC-CM on the expression of proliferation marker (PCNA), EMT markers (Snail, Vimentin, E-cadherin, N-cadherin) in heat-exposed residual HCC cells were markedly reversed (Fig. 2a, b). On the other hand, administration of exogenous recombinant POSTN to heat-treated HCC residual cells resulted in the increased expression of proliferation markers (Ki-67, cyclin D1 and PCNA) and the induced changes of EMT markers (Snail, Vimentin, E-cadherin and N-cadherin) and showing a spindle-shaped appearance (Fig. 2c–f). These data indicate that POSTN plays a major role in the tumor-promoting effects imposed by HSC-CM.

### POSTN induces the activation of p52Shc/ERK1/2 in heat-treated residual HCC cells

**Fig. 3:**
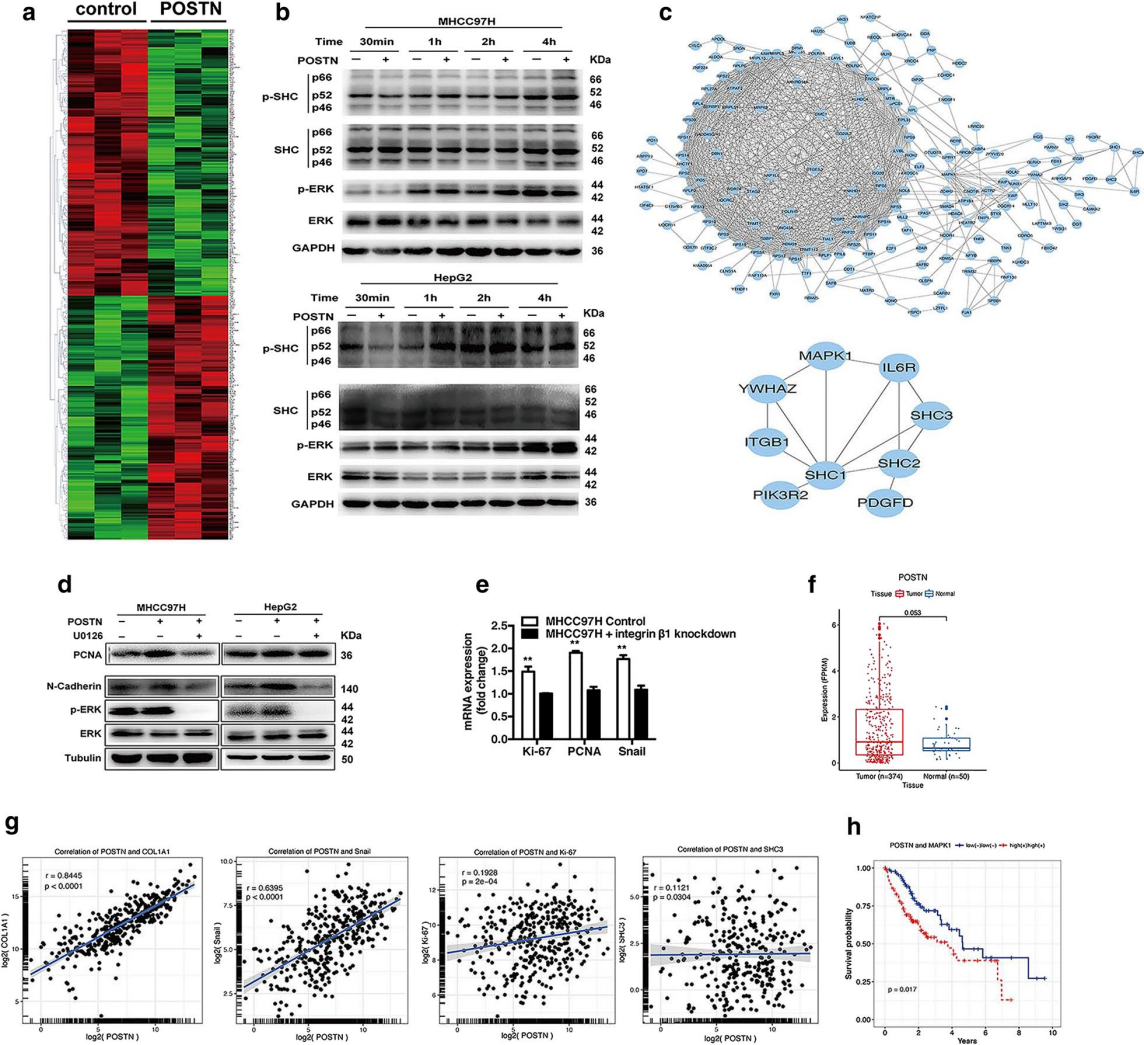
POSTN induced the Shc-ERK activation of heat-exposed residual HCC cells through integrin β1. **a** The mRNA expression profile of heat-treated residual MHCC97H cells in response to POSTN was illustrated as a heatmap. Red, green represent high and low mRNA expression. **b** With POSTN treatment, the phosphorylated of p52Shc and ERK1/2 in heat-exposed residual HCC cells (MCHCC97H and HepG2) were significantly increased in a time-dependent manner. **c** PPI network analysis of the differentially expressed genes identified Shc as a gene of biological importance in POSTN-mediated signaling networks and a diagram illustrated the interaction of Shc with the molecules (e.g., ITGB1 and MAPK1). **d** When heat-exposed residual HCC cells (MCHCC97H and HepG2) were treated with POSTN, the levels of PCNA, N-cadherin and ERK1/2 phosphorylation were significantly increased. ERK1/2 inhibitor (U0126, 25 μM) reversed the above POSTN-induced increase. **e** With the stimulation of exogenous POSTN, the levels of Ki-67, PCNA and Snail mRNA expression were significantly decreased in heat-exposed residual integrin β1-knockdown MHCC97H cells. **f** Expression of POSTN in HCC tissues (n = 374) than that of adjacent non-tumor tissues (n = 50) in the HCC data of TCGA cohorts. **g** A significant positive correlation between the degree of POSTN expression also showed with that of COL1A1 (r = 0.8445, P < 0.0001), Ki-67 (r = 0.1928, P = 2×10^−4^), Snail (r = 0.6395, P < 0.0001), and Sch3 (r = 0.1121, P = 0.0304) in the TCGA-HCC cohorts. **h** HCC patients were stratified by POSTN and MAPK1 (ERK2) expression and the co-expression of POSTN and ERK2 predicted poor-survival prognosis in the TCGA-HCC cohorts by Kaplan–Meier analyses

To delineate the mechanism by which POSTN promotes the progression of residual HCC, we performed microarray experiments by analyzing heat-treated residual HCC cells cultured with POSTN. In heat-treated residual MHCC97H cells, 360 genes whose expression was significantly modulated (P < 0.05; twofold change) by the presence of POSTN, including the upregulation of master genes involved in proliferation (e.g., PIBF1, ANKHD1 and RIOK2) and EMT (e.g., ARHGAP5 and HMG20B) (Fig. 3a). Importantly, PPI network of the differentially expressed genes revealed that Shc was probably a gene that of biological importance in POSTN-mediated signaling network, which linked integrin β1 and MAPK (Fig. 3c). Moreover, differentially expressed Shc in the Microarrays (upregulated ~ threefold upon POSTN treatment) was confirmed by western blot. As shown in Fig. 3b, phosphorylated p52Shc expression was markedly increased in a time-dependent manner whereas the p46Shc or p66Shc isoform was not significantly affected. This was paralleled by enhanced expression of phosphorylated Erk1/2. POSTN induced the activation of ERK1/2 in heat-treated HCC residual cells and increased the expression of PCNA and N-cadherin whereas ERK inhibitor abolished POSTN-induced ERK phosphorylation and the upregulation of PCNA and N-Cadherin (Fig. 3d). As previously described, POSTN promotes tumor development through integrin receptors [30]. POSTN-induced expression of proliferation and EMT (PCNA, Ki-67, Snail) was significantly blunted in MHCC97H cells with integrin β1 knockdown (Fig. 3e). These data suggest that POSTN promotes malignant behaviors of heat-treated residual HCC cells via integrin β1 and p52Shc/ERK1/2 pathway.

To validate the in vitro results regarding the regulatory axis of POSTN, we analyzed the correlation between the degree of POSTN and that of COL1A1, Ki-67, Snail and Shc expression in gene expression profiles from HCC data from TCGA. The queried data revealed a higher level of POSTN in HCC tissues (n = 374) than that of adjacent non-tumor tissues (n = 50) (*P *= 0.053) (Fig. 3f). Furthermore, the degree of POSTN expression also showed a significant positive correlation with that of collagen-1 (r = 0.8445, *P *< 0.0001), Ki-67 (r = 0.1928, *P *= 2 × 10^−4^), Snail (r = 0.6395, *P *< 0.0001), and Sch3 (r = 0.1121, *P *= 0.0304) in TCGA-HCC cohorts (Fig. 3g). Previous study showed that MAPK/ERK pathway could be activated by Shc3. When HCC patients were categorized according to POSTN and MAPK1 (ERK2) expression, the co-expression of POSTN and ERK2 predicted poor-survival prognosis in TCGA-HCC cohorts (P = 0.017) (Fig. 3h). These results suggest that POSTN expression is closely associated with HSC activation, and the expression of proliferation, EMT and Shc/ERK-mediated signaling activities in HCC tissues.

### Activated HSCs promote the in vivo tumor progression of heat-treated residual HCC cells

**Fig. 4:**
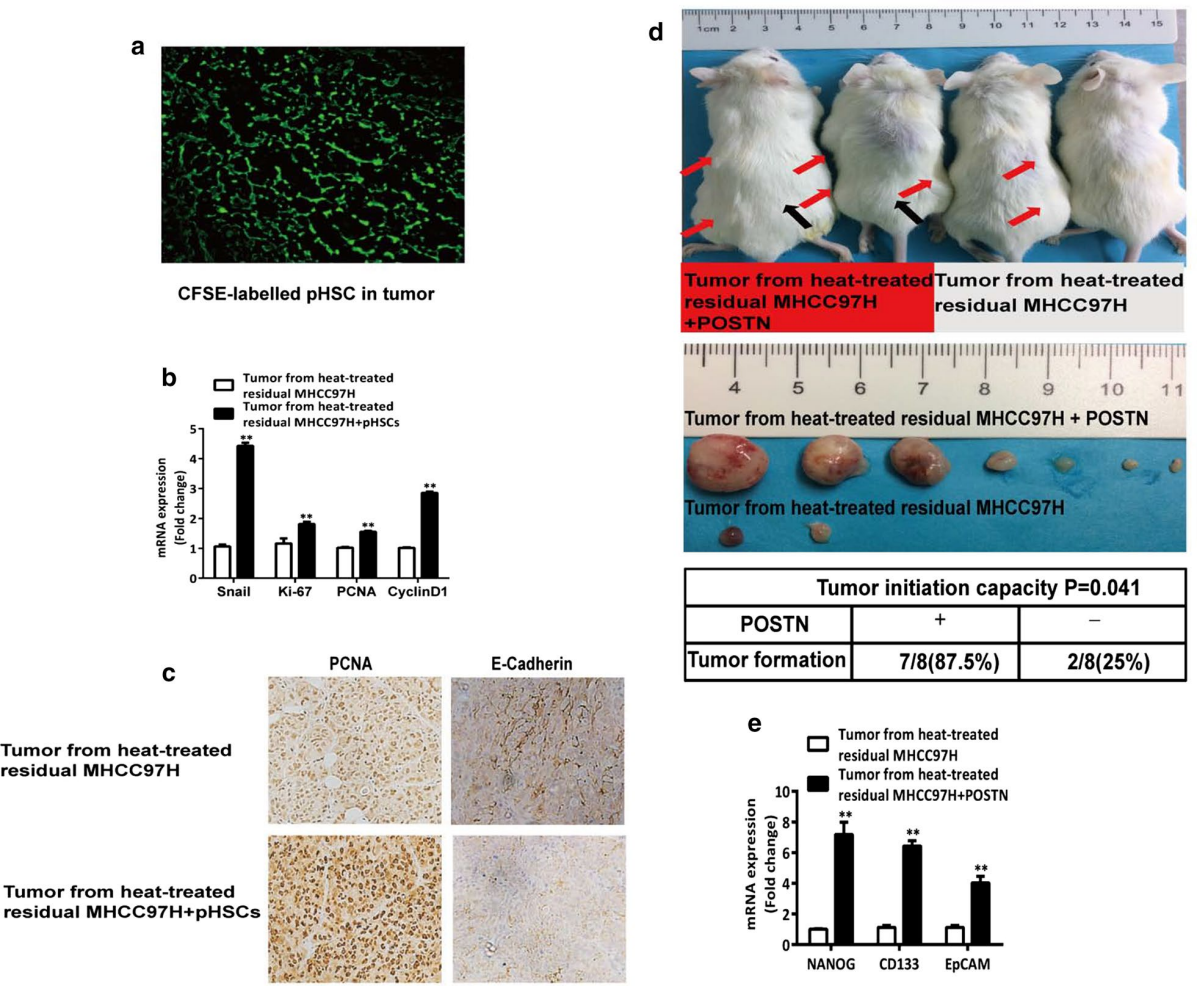
Activated HSCs promoted the in vivo tumor progression of heat-treated residual HCC cells. **a** HSCs cells were found in the tumors at 2 weeks after the inoculation of heat-treated residual MHCC97H cells with CFSE-labelled pHSCs. **b**, **c** Up-regulation of Ki-67, PCNA, cyclin D1 and Snail mRNA expression was found in the tumors formed from heat-exposed residual MHCC97H and pHSCs compared with the tumors from heat-exposed residual MHCC97H alone, as detected by qRT-PCR. The protein expression of PCNA and E-Cadherin was shown by immunohistochemical analysis. **d** Xenograft tumorigenicity assay. Heat-exposed residual MHCC97H (2 × 10^4^ cells) with 100 ng/mL POSTN subcutaneously inoculated into NOD/SCID mice developed more and larger tumors compared to the cells injected alone. Red arrows on the left flank represent the tumors formed by heat-exposed residual MHCC97H cells with 100 ng/mL POSTN while black arrows on the right flank represent the tumors formed by heat-exposed residual MHCC97H cells alone. The number and images of tumors developed in mice after 8 weeks are shown. The two groups were compared by using Fisher exact test. **e** The mRNA expression of NANOG, CD133, EpCAM was significantly up-regulated in the tumors generated from heat-exposed residual MHCC97H cells with 100 ng/mL POSTN. **P < 0.01; *P < 0.05

In our previous study [26], heat-treated residual HCC cells with activated HSCs inoculated subcutaneously into nude mice grew faster compared with residual HCC cells inoculated alone. To verify the presence of activated HSCs in the tumors, CFSE-labelled HSCs were found present in the tumor tissue (Fig. 4a). Using the same batch of tumor specimens from our previous work [26], the levels of PCNA, Ki-67, cyclin D1, Snail expressions were significantly higher and E-cadherin expression was markedly lower in the tumors from heat-treated residual HCC cells with activated HSCs than the tumors formed by heat-treated residual HCC cells alone (Fig. 4b, c). To analyze the effect of POSTN on the tumorigenicity, we injected a limited number of heat-treated residual HCC cells subcutaneously into NOD/SCID mice with or without POSTN. After 2 months, heat-treated residual HCC cells injected with POSTN developed more and larger tumors compared to the cells injected alone (7/8 vs 2/8, *P *= 0.041) (Fig. 4d). The levels of Nanog, CD133, and EpCAM mRNA expression were significantly increased in the tumors from heat-treated residual HCC cells with POSTN compared to the tumors formed by heat-treated residual HCC cells alone (Fig. 4e). These results suggest that activated HSCs promote the in vivo progression of heat-treated residual HCC cells through increasing proliferation, EMT and POSTN-enhanced tumorigenicity.

### Vitamin D receptor (VDR) mediate transrepression in the presence of VDR ligand calcipotriol to inhibit the expression of POSTN

**Fig. 5:**
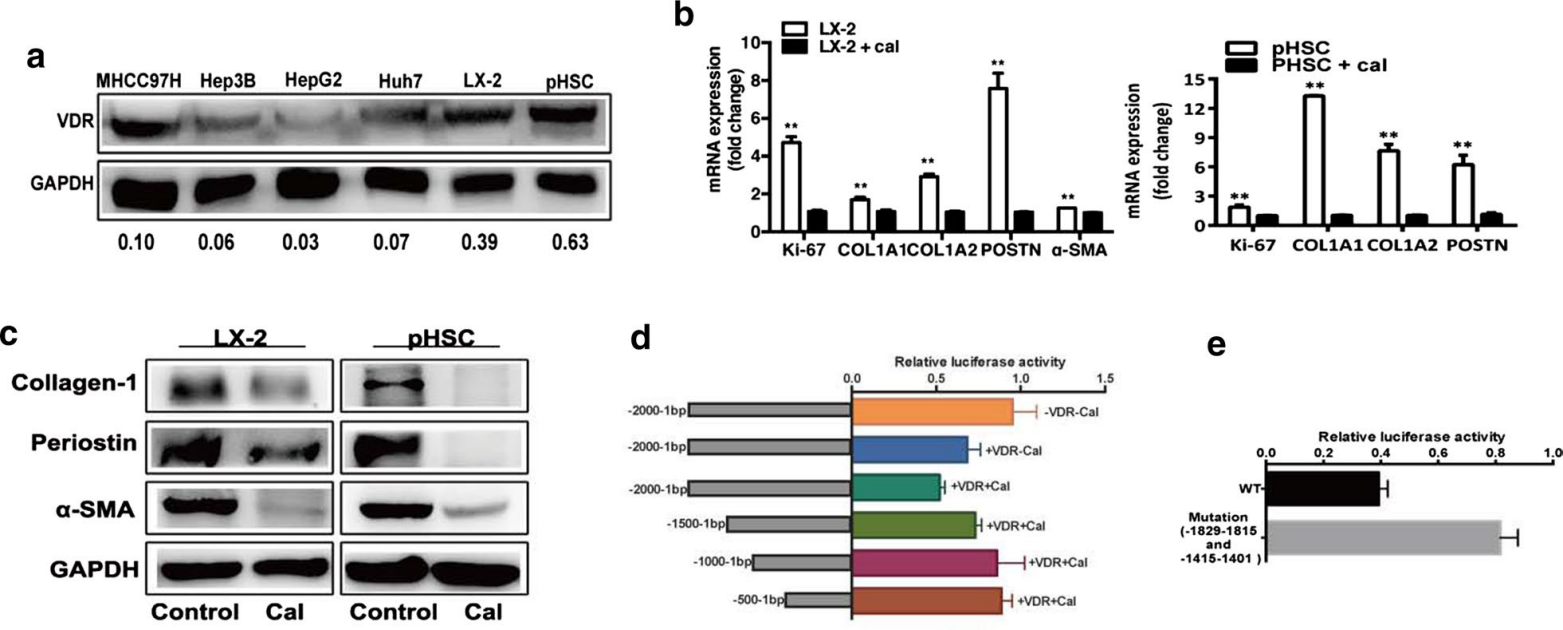
VDR regulates the expression of POSTN in presence of calcipotriol. **a** Vitamin D receptor (VDR) expression in activated HSCs (LX2 and pHSC) and HCC cell lines (MHCC97H, Hep3B, HepG2 and Huh7) was detected by Western blot. **b** The markers and activities of activated HSCs (α-SMA, COL1A1, COL1A2, Ki-67, and POSTN) were down-regulated in LX2 cells and pHSC cells by calcipotriol treatment, as analyzed using qRT-PCR. **c** Protein expression of α-SMA, POSTN and collagen-1 were reduced in LX2 cells and pHSC cells by calcipotriol treatment, as measured by western blot. **d** Results of Luciferase reporter assay for POSTN promoter constructs. A ~ 2-kb-long POSTN promoter and its serial deletion constructs were measured in HEK 293T cells and results of the firefly luciferase were normalized to the Renilla luciferase activity. **e** Luciferase activity of mutant reporter plasmids that the two putative VDR transcription factor binding sites were altered by site-directed mutagenesis in the full-length POSTN promoter. Calcipotriol, Cal. VDR, VDR-expressing plasmid. **P < 0.01; *P < 0.05

Vitamin D contributes to preventing the development and progression of cancers [32]. The most active form of vitamin D, calcipotriol, reduces tissue fibrosis and suppresses tumor progression by inhibiting the activation of HSCs and pancreatic stellate cells as a VDR agonist [33–35]. The protein expression level of VDR in activated HSCs (LX2 and pHSCs) was found to be significantly increased in comparison to HCC cell lines (MHCC97H, Hep3B, HepG2, Huh7) (Fig. 5a).

TGF-β1 is the most profibrotic factor in liver disease. Calcipotriol suppressed the activities of HSCs stimulated by TGF-β1, as indicated by downregulating the markers of representing the activities of HSCs (α-SMA, Ki-67, PCNA, COL1A1, COL1A2) (Fig. 5b, c). Following treatment with VDR ligand calcipotriol, POSTN expression was decreased in HSCs. As shown in Fig. 5d, VDR mediated transrepression of the POSTN promoter activity in presence of calcipotriol. To determine the binding sites of VDR in POSTN promoter that is important for POSTN expression, we constructed luciferase reporter plasmids of approximately 2000 kb in length (− 2000 bp from −1 bp relative to the transcription start site) and serial deletion constructs. When measuring the promoter activity of the constructs in HEK 293T cells in the presence of calcipotriol, we found it to be significantly increased at two segments: between − 2000 and − 1500 and between − 1500 and − 1000, suggesting that these two promoter segments are important for POSTN regulation (Fig. 5d). And mutant construct for the VDR binding sites were generated using site-specific mutagenesis. The mutant of the − 1829 to − 1815 and − 1415 to − 1401 VDR binding sites revealed a significant increase in activity (Fig. 5e). These results suggest that VDR as a member of ligand-induced transcription factors mediate transrepression in the presence of VDR ligand calcipotriol to regulate the expression of POSTN.

### Calcipotriol suppresses the activated HSCs-enhanced progression of heat-treated residual HCC cells

**Fig. 6:**
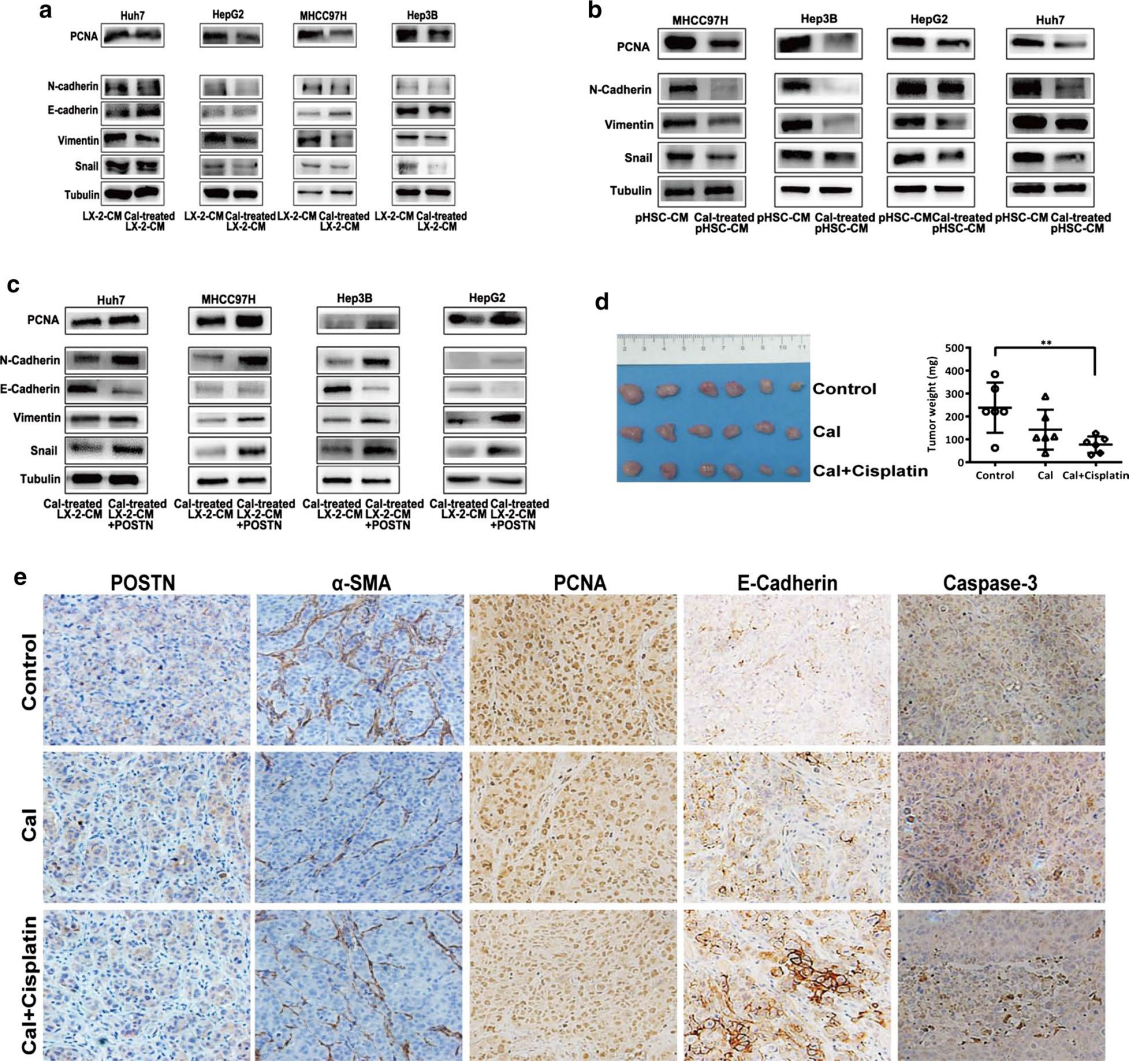
Calcipotriol suppressed the activated HSCs-induced tumor progression of heat-treated residual HCC cells in vivo. **a**, **b** The changes of Snail, Vimentin, E-cadherin and N-cadherin, PCNA expression in heat-exposed residual HCC cells (MHCC97H, Hep3B, HepG2 and Huh7) cultured with conditioned medium (CM) from HSCs or calcipotriol-treated HSCs were compared. HSCs cells (LX2 and pHSC) were stimulated by the pre-treatment with TGF-β1. **c** The changes of Snail, Vimentin, E-cadherin and N-cadherin, PCNA expression in heat-treated residual HCC cells were reversed when CM from calcipotriol-treated LX2 cells was supplemented with 100 ng/mL POSTN. **d** Calcipotriol inhibited tumor growth compared with the control group. The combination therapy of calcipotriol and cisplatin significantly reduced tumor growth. **e** The expression of POSTN, α-SMA, PCNA, E-Cadherin and cleaved-Caspase-3 was detected by immunohistochemical analysis. Calcipotriol, Cal. **P < 0.01; *P < 0.05

To validate whether calcipotriol could block unfavorable effects of activated HSCs on heat-treated residual HCC cells, we collected conditioned media (CM) from calcipotriol-treated HSCs. CM from calcipotriol-treated HSCs less upregulated the expression of PCNA and EMT markers (Snail, Vimentin, N-cadherin) in heat-exposed residual HCC cells compared with CM from calcipotriol-untreated HSCs (Fig. 6a, b). These effects were recovered when CM from calcipotriol-treated HSCs was supplemented with 100 ng/mL POSTN (Fig. 6c). These data indicate that calcipotriol could inhibit the pro-tumorigenic effects of activated HSCs on heat-exposed residual HCC cells through suppressing POSTN secretion.

To explore whether deactivating HSCs could suppress the in vivo tumor progression of heat-treated residual HCC cells, we assessed the therapeutic potential of calcipotriol. Mice bearing tumors derived from heat-exposed residual HCC cells and activated HSCs were randomly assigned into three treatment groups. An apparent tendency towards calcipotriol alone inhibiting tumor growth compared with the control group was observed, although it did not reach statistically significant. Since HCC cells expressed low VDR expression, the observed therapeutic effect of calcipotriol would likely result from inhibiting activated HSCs. Importantly, with cisplatin additional treatment, tumor growth was further markedly reduced (Fig. 6d), suggesting cisplatin has an addition to the inhibitory effect of calcipotriol on heat-treated residual HCC. In agreement with the reduction of tumor volume, the significantly decreased expression of POSTN, α-SMA and PCNA, and the apparently increased expression of E-cadherin and cleaved-Caspase-3 were observed (Fig. 6e). These data suggest that calcipotriol plus cisplatin suppresses the activated HSCs-induced tumor progression of heat-treated residual HCC cells via the inhibited POSTN expression and the increased apoptosis.

## Discussion

Local ablation, preferably RFA, is currently considered as the standard and first-line treatment option for patients with unresectable early-stage HCC [1, 2]. However, when it is expanded to the treatment of medium and large lesions, high local recurrence accounts for the worse prognosis due to incomplete ablated tumors [4, 5]. Recently, accelerated tumor progression after incomplete RFA has been increasingly reported [8–10, 13, 36]. In this study, we firstly demonstrate that activated HSCs promote the tumor progression of residual HCC after sublethal heat treatment through the release of POSTN. Second, we show that this detrimental effect could be reversed by vitamin D analogue calcipotriol, and cisplatin has an addition to the inhibitory effect of calcipotriol on heat-treated residual HCC, suggesting that calcipotriol plus cisplatin could be applied to thwart the accelerated progression of residual HCC after suboptimal heat treatment. Our findings have clinical implications in improving the survival outcome of RFA.

In contrast to previous studies focusing on the changes of HCC cells response to heat treatment [14, 18, 19, 37–39], we provide a new evidence that activated HSCs enriched in tumor-promoting microenvironment could promote the accelerated progression of heat-treated residual HCC through POSTN secretion. POSTN is one of pivotal proteins from activated HSCs and becomes a better anti-fibrotic therapeutic target [40]. It is also involved in initiating the process of proliferation and EMT in cancer cells [30]. In addition, stromal expression of POSTN is associated with high aggressiveness of HCC [41]. To our knowledge, there is no study of the interaction between activated HSCs and post-treatment residual HCC via POSTN.

Similar to the function of fibroblasts in the process of wound healing [42], activated HSCs are recruited into the peri-ablative zone after RFA [25]. Therefore, it is plausible to explore the cross-talks between activated HSCs and heat-treated residual HCC cells as a tumor promoting mechanism. In the present study, we showed that POSTN from activated HSCs promoted the proliferation, motility, invasion prominent activation of EMT as well as decreased apoptosis in heat-exposed residual HCC cells. The result is consistent to the previous reports of POSTN accelerating the progression in other tumors [43, 44]. Moreover, using gene microarrays, we identified Shc as a molecule with biological importance in POSTN-mediated signaling networks. Further analysis of data from TCGA revealed POSTN expression was strongly associated with markers of HSC activation, proliferation, EMT and Shc-mediated signaling, and the co-expression of POSTN and ERK2 conferred poor-survival prognosis in the TCGA-HCC cohort. ERK1/2 are known direct downstream mediator of Shc. ERK activation has been involved in the regulation of proliferation and EMT of HCC cells [14, 37, 38]. Recently, it has been reported that increased Shc3 expression results in activation of MEK/ERK in HCC independently of c-Raf [45]. Shc is signaling partner for integrins [46–48]. In this study, we demonstrated that POSTN promoted the malignant behaviors of heat-exposed residual HCC cells via integrin β1 and p52Shc-ERK1/2 activation, indicating that integrin β1-Shc-ERK axis as major responsible pathway for delivering signals from POSTN to heat-exposed residual HCC cells.

Considering the important roles of activated HSCs on the accelerated growth of heat-exposed residual HCC cells, we sought to revert activated HSCs to quiescence or block the POSTN secretion, which might help prevent the progression of heat-treated residual HCC cells. Vitamin and its analogues have been shown to attenuate fibrosis and delay tumor progression through suppressing the activities of fibroblasts, stellate cells [33–35]. In scleroderma, the vitamin D analogue inhibits TGF β-induced POSTN expression to reduce fibrosis [49]. Another study has reported that calcipotriol is capable of inhibiting POSTN expression in pancreatic ductal adenocarcinoma [33]. In line with the above studies, calcipotriol significantly reduced POSTN secretion from activated HSCs and inhibited the subsequent pro-tumorigenic effects of activated HSC on heat-exposed residual HCC cells. The two VDR transcription factor binding sites in the POSTN promoter was identified as crucial negative regulators of POSTN expression in HSCs, suggesting that POSTN expression in HSCs is regulated by calcipotriol via the VDR axis. Because VDR was highly expressed on the HSCs relative to HCC cells, the observed therapeutic effect of calcipotriol inhibiting tumor growth likely results from suppressing activated HSCs. In this study, calcipotriol plus cisplatin showed the additive and therapeutic effects, suggesting combination strategies could be used to suppress the accelerated progression of residual HCC after insufficient heat treatment.

This study has several limitations. First, although we demonstrate the role of POSTN in tumor progression of heat-treated residual HCC, we could not exclude the other factors implicated in post-inflammation reaction after RFA that will promote tumor progression of heat-treated residual HCC, such as a Th1 cytokine pattern, cellular infiltration at the periablational zone, heat shock proteins. Second, it is better to have a group of tumor-bearing mice treated with cisplatin alone in experimental therapy. However, we showed that conventional anticancer agent cisplatin had an addition to the inhibitory effect of calcipotriol on heat-treated residual HCC. Cisplatin is a common therapeutic agent used for chemotherapy in HCC and has been shown to have tumor inhibition in the HCC xenografts [50]. Whether there exists a synergistic relationship between calcipotriol and cisplatin needs to be verified in additional animal studies. Third, we employed a subcutaneous tumor model of implanting heat-treated residual HCC cells and activated HSCs in mice to assess the response to experimental therapy. Better animal models (an orthotopic model of HCC, rabbit VX2 hepatoma) are needed to verify our findings. Fourth, calcipotriol treatment carries risks of local and systemic side effects because of its toxicity. Topical application of calcipotriol or the use of cholecalciferol as an alternative to calcipotriol may be a safe and effective therapy.

## Conclusions

**Fig. 7:**
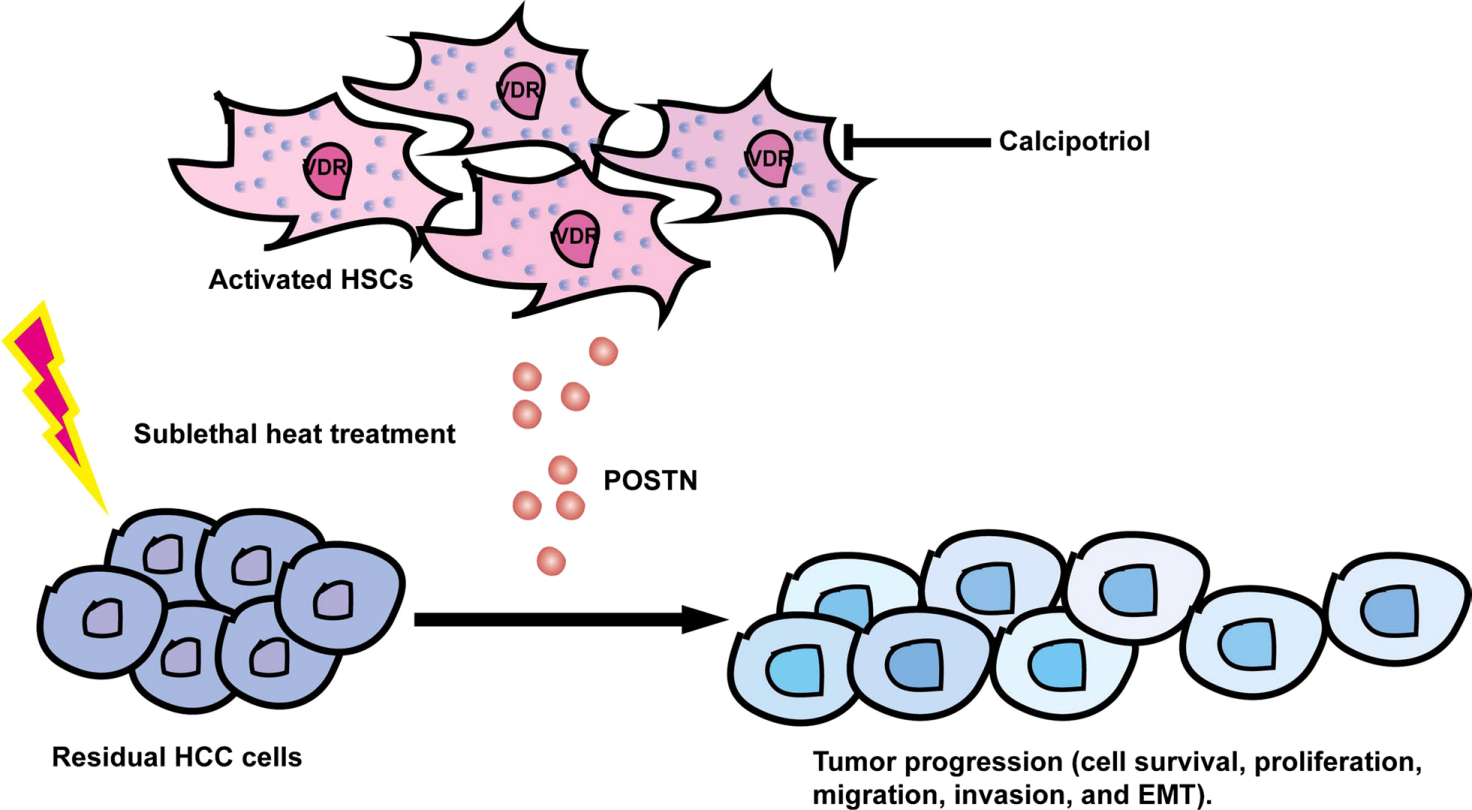
A proposed graphical diagram that activated HSCs promote tumor progression of heat-treated residual HCC cells via POSTN secretion which could be inhibited by calcipotriol

In conclusion (Fig. 7), our study reveals that activated HSCs promote the tumor progression of residual HCC after sublethal heat treatment by releasing POSTN that could be inhibited by calcipotriol, and proposes calcipotriol plus cisplatin as a new therapeutic potential to inhibit the incomplete RFA-accelerated progression of residual HCC.

## Additional file

**Additional file 1: Table S1.** Primers for quantitative RT-PCR.

## Abbreviations

HCC: hepatocellular carcinoma
HSCs: hepatic stellate cells
CM: conditioned medium
ERK1/2: extracellular signal-related kinase 1 and 2
TGF-β1: transforming growth factor-β1
EMT: epithelial–mesenchymal transition
RFA: radiofrequency ablation
POSTN: periostin
VDR: vitamin D receptor
SD: standard deviation
CFSE: 5,6-carboxyfluorescein diacetate succinimidylester
