# Supplementary material for: Periostin involved in the activated hepatic stellate cells-induced progression of residual hepatocellular carcinoma after sublethal heat treatment: its role and potential for therapeutic inhibition
Source: J Transl Med. 2018 Nov 6;16:302. doi: 10.1186/s12967-018-1676-3 (PMC6219107; doi:10.1186/s12967-018-1676-3)
Supplement: Supplementary file 1 — Additional file 1: Table S1. Primers for quantitative RT-PCR. [file 12967_2018_1676_MOESM1_ESM.docx]

**Table S1 Primers for quantitative RT-PCR**

| Gene | Sequence |
| --- | --- |
| PCNA | Forward-5’-ACACTAAGGGCCGAAGATAACG-3’  Reverse-5’-ACAGCATCTCCAATATGGCTGA-3’ |
| Ki-67 | Forward-5’-ACGCCTGGTTACTATCAAAAGG-3’  Reverse-5’-CAGACCCATTTACTTGTGTTGGA-3’ |
| CyclinD1  POSTN | Forward-5’- GCTGCGAAGTGGAAACCATC-3’  Reverse-5’- CCTCCTTCTGCACACATTTGAA-3’  Forward-5’-GACCGTGTGCTTACACAAATTG-3’  Reverse-5’-AAGTGACCGTCTCTTCCAAGG-3’ |
| Snail | Forward-5’-TTTACCTTCCAGCAGCCCTA-3’  Reverse-5’-GACAGAGTCCCAGATGAGCA-3’ |
| COL1A1 | Forward-5’-AGAGGAAGGAAAGCGAGGAG-3’  Reverse -5’-GGACCAGCAACACCATCTG-3’ |
| COL1A2 | Forward-5’- AGGTGGAAAAGGTGAACAGG-3’  Reverse -5’-AGGACCAGGGAGACCAAACT-3’ |
| α-SMA | Forward-5’- ATGTGCGACGAAGACGAGA-3’  Reverse -5’- TTCTGACCCATACCGACCAT-3’ |
| NANOG | Forward-5’-AGGCAAACAACCCACTTCTG-3’  Reverse-5’-TCTGCTGGAGGCTGAGGTAT-3’ |
| CD133 | Forward-5’-GCGATCAAGGAGACCAAAGA-3’  Reverse -5’-GACCGCAGGCTAGTTTTCAC-3’ |
| EpCAM  GAPDH  β-actin | Forward-5’-ATCCTGACTGCGATGAGAGC-3’  Reverse -5’-TGTCCTTGTCTGTTCTTCTGACC-3’  Forward-5’-GGAGCGAGATCCCTCCAAAAT-3’  Reverse-5’-GGCTGTTGTCATACTTCTCATGG-3’  Forward-5’-CATGTACGTTGCTATCCAGGC-3’  Reverse-5’-CTCCTTAATGTCACGCACGAT-3 |
